# Bisphosphonate-related osteonecrosis. Application of adipose-derived stem cells in an experimental murine model

**DOI:** 10.4317/medoral.22959

**Published:** 2019-06-25

**Authors:** Estefanía Alonso-Rodriguez, Javier González-Martín-Moro, José-Luis Cebrián-Carretero, José-Luis Del Castillo, Jose-Juan Pozo-Kreilinger, Elena Ruiz-Bravo, Mariano García-Arranz, Juan Hernández-Godoy, Miguel Burgueño

**Affiliations:** 1 M.D. Department of Oral and Maxillofacial Surgery, Hospital Universitario La Paz, Paseo de la Castellana 261, 28046 Madrid, Spain. Chief of Department: Miguel Burgueño; 2 M.D, Ph.D. Department of Oral and Maxillofacial Surgery, Hospital Universitario La Paz, Paseo de la Castellana 261, 28046 Madrid, Spain. Chief of Department: Miguel Burgueño; 3 M.D. Department of Pathology, Hospital Universitario La Paz, Paseo de la Castellana 261, 28046 Madrid, Spain; 4 Department of Surgery, Faculty of Medicine, Universidad Autónoma de Madrid, C/ Arzobispo Morcillo s/n, 28029 Madrid, Spain; 5 Cell Therapy Laboratory, Instituto de Investigación Sanitario Fundación Jiménez Díaz, Avda. Reyes Católicos 2,28040 Madrid, Spain; 6 M.D. Plastic Surgery. Private practice

## Abstract

**Background:**

Bisphosphonate-related osteonecrosis of the jaw is a pathological condition without effective established treatment and preventive strategies. The aim of this study was to analyse the effect of adipose-derived stem cells (ASC) in an experimental murine model of osteonecrosis.

**Material and Methods:**

38 Wistar rats were injected intraperitoneally with zoledronic acid. After treatment, upper jaw molars were extracted. The animals were randomly assigned to one of two groups. In the control group, saline solution was applied over the alveolar sockets after the tooth extractions. In the treatment group, ASCs were applied instead of saline solution. The control and treatment groups were subdivided based on the time of euthanasia. A clinical and histological analysis was performed.

**Results:**

The presence of osteonecrosis in alveolar bone was observed in a similar distribution in both groups. In the ASC-treated group, new bone formation was greater than in controls.

**Conclusions:**

In this study, application of ASCs showed greater new bone formation in an osteonecrosis-like murine model. Previous inhibited post-extraction bone remodelling could be reactivated, and these findings appeared to be secondary to implantation of ASCs.

** Key words:**Osteonecrosis; bisphosphonates, Mesenchymal stem cells (MSC), adipose-derived stem cells (ASCs), zoledronic acid.

## Introduction

Medication-related osteonecrosis of the jaw (MRONJ) is a painful condition that severely affects quality of life. The main drugs associated with MRONJ are bisphosphonates (BPs), which are antiresorptive drugs used to treat many diseases and conditions of increased bone resorption such as osteoporosis, Paget’s disease, multiple myeloma, hypercalcaemia of malignancy or osteolytic bone metastases, among others.

Osteonecrosis of the jaw is a pathological condition of increasing frequency with a poorly understood pathophysiology. Despite all of the studies and advances achieved, MRONJ still lacks effective treatment and preventive strategies. The main hypotheses regarding the pathophysiology of osteonecrosis are pharmacological inhibition of bone remodelling and angiogenesis, the presence of trauma and/or inflammation/infection, soft tissue BP toxicity, and an innate or acquired immune dysfunction(1,2). Given the variety of hypotheses, there are many treatments but none is totally successful. Due to the lack of curative treatment for MRONJ, one of the best strategies nowadays is to avoid it.

Mesenchymal stem cells (MSC) have recently been studied for the treatment of MRONJ, and they appear to be beneficial for this issue. However, there are few published studies. Some are case reports and most use MSCs isolated from bone marrow (BM-MSC) (3-9). Studies on prevention of MRONJ and stem cells are emerging (4,10,11) but they are scarce, and this field needs to be investigated.

Adipose tissue is an abundant and easily accessible source of MSCs, known as adipose-derived stem cells (ASCs). The capacity of ASCs to promote angiogenesis, secrete growth factors, regulate the inflammatory process, and differentiate into multiple cell types makes them a potentially ideal therapy for chronic wounds (12). These properties make ASCs very attractive as a possible tool for prevention of MRONJ.

The aim of this study was to analyse the potential effect of ASCs in an experimental murine model of BRONJ (bisphosphonate-related osteonecrosis of the jaw)-like disease induced with zoledronic acid (ZA) after tooth extraction without other enhancer drugs.

## Material and Methods

-Study design

Currently, there is no standard MRONJ animal model. A previously published MRONJ-like disease model(13) has been followed with some variations. The present study was approved by the Ethics Committee for Animal Care of La Paz University Hospital, Madrid, Spain (CEI 69-1212-A131).

Thirty-eight Wistar female and two male rats (Charles River, France) with an average age of 8 weeks and an average weight of 275 grams were used. All the animals were kept in a controlled-temperature environment with food and water ad libitum.

All the female animals were injected intraperitoneally with zoledronic acid (ZA) (Sandoz, 4 mg/100 ml) at dosages of 0.1 mg ZA/kg three times per week for 9 weeks. The males received no treatment, they were used just to obtain adipose tissue. After this treatment, three right upper molars were extracted from all the animals under general anaesthesia with the objective of establishing the osteonecrosis model.

**Figure 1:**
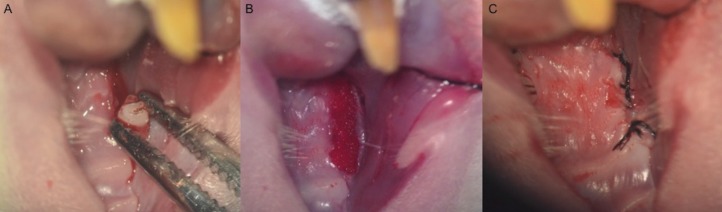
A: Tooth extraction with optical microscope assistance; B: haemostatic sponge with ASCs over the alveolar sockets; C: advanced buccal mucoperiosteal flap.

The molars were extracted with under optical microscope assistance. After syndesmotomy with a spatula, the molars were luxated with a spatulated osteotrimmer and a mosquito clamp was used as forceps (Fig. 1A). Surgical milling of the alveolar bone was carried out with a tungsten bur without irrigation to add more trauma.

The animals were randomly assigned to one of two groups. In the control group, saline solution was applied over the alveolar sockets after the tooth extractions. In the treatment group, ASCs were applied instead of saline solution (one million allogeneic cells in 25 microlitres of Ringer’s lactate solution in each maxilla). An absorbable haemostatic gelatine sponge (Spongostan®) was used as a carrier for ASCs or for saline solution in each group (Fig. 1B). An advanced buccal mucoperiosteal flap to cover the alveolar bone post-extraction was performed for all the animals. Gingival borders were attached with a continuous suture covering the defect without tension (Fig. 1C).

After tooth extractions, the animals were decapitated under general anaesthesia (inhaled isoflurane with oxygen 3 L/min) and the heads were harvested for histological study. The control and treatment groups were subdivided as follows based on the time of euthanasia:

- Group 1: 6 control rats, euthanised 1 month after tooth extraction;

- Group 2: 8 treatments rats, euthanised 1 month after tooth extraction;

- Group 3: 6 control rats, euthanised 2 months after tooth extraction;

- Group 4: 9 treatments rats, euthanised 2 months after tooth extraction.

Tramadol was administered once preoperatively, and daily subcutaneously for 3 postoperative days.

Intraperitoneal general anaesthesia (ketamine 100 mg/kg + diazepam 8 mg/kg + atropine 0.4 mg/kg) was first used; however, 9 rats (all controls) died during the procedure or on the postoperative days. Isoflurane was then used with no further deaths until euthanasia.

-ASC preparation

Allogeneic ASCs were obtained under inhaled general anaesthesia from the subcutaneous abdominal and inguinal fat tissue of the two male Wistar rats. This process is based on separation by centrifugation, use of type I collagenase and the plastic adhesion properties of the cells.

Fat tissue was digested with type I collagenase (0.075%; Gibco BRL, Paisley, Scotland, UK). The collagenase was then inactivated by addition of an equal volume of Dulbecco’s Modified Eagle Medium (DMEM; Gibco BRL), which contained 10% foetal bovine serum (FBS; Gibco BRL). The digested tissue was centrifuged at 300 G for 10 min. After filtering through a 70-µm nylon mesh, the cells were cultured in DMEM (Gibco BRL) containing 10% FBS (Gibco BRL) and 1% penicillin-streptomycin (Gibco BRL). The medium was changed to remove nonadherent cells 24 h after seeding and every 3–4 days thereafter. When 80%–90% confluence was achieved, the cells were detached with 0.05% (v/v) trypsin-ethylenediaminetetraacetic acid (Gibco BRL) in phosphate-buffered saline. The subcultured cells were frozen until the week before their use.

-Histological analysis 

Histological features are not included in the American Association of Oral and Maxillofacial Surgeons’ definition of MRONJ in humans. However, in rats, this description is one of the main tools for studying osteonecrosis. In this study, the characteristics of osteonecrosis of the jaw described previously in the literature have been followed (14,15). Histological sections were stained with haematoxylin-eosin, periodic acid-Schiff and Masson’s trichrome and the analysis was performed by two pathologists in a blinded fashion (light microscopy, 40x magnification). The parameters evaluated were as follows:

1. Osteonecrosis: defined as a portion of bone in which greater than or equal to 10 adjacent empty osteocyte lacunae are present, as previously described by Yamashita *et al.* (16), in 3 different random cuts (20x).

2. Osteoclast count: Arithmetic mean of the count in three different fields (40x), in two different random cuts in the alveolar process of the upper jaw.

3. Vascularisation: graded by a visual scale from 1 to 5 (1: absence of vessels; 5: duplication of the vascular surface area compared with this area in the controls).

4. Degree of alveolar remodelling (after tooth extraction): A scale was established from 0 to 4 (0: absence of bone resorption; 1: < 25%; 2: 25-50%; 3: 50-75%; 4: 75-100% of alveolar bone area resorbed and substituted by fibrous tissue).

5. Bone neoformation: New bone formation graded on a scale from 0 to 4 (0: absence of bone formation; 1: < 25%; 2: 25-50%; 3: 50-75%; 4: 75-100% of bone formation).

6. Inflammatory infiltrates: Evaluated as presence or absence.

-Statistical analysis

The data analyses were performed with SPSS v. 20. A statistical significance level of *p*<.05 was used.

The quantitative variables were analysed with the nonparametric Kruskal–Wallis test or the Mann–Whitney U test if necessary.

The qualitative variables were analysed with the χ2 test or Fisher’s exact test (if N<20 or if any expected value is less than 5). When the χ2 test was used, Yates’s correction was applied.

## Results

None of the animals had exposed bone at the extraction sites.

-ASCs versus controls 

When all the ASC-treated rats (groups 2 and 4) and all the control rats (groups 1 and 3) were compared ( Table 1), all showed the presence of osteonecrosis in alveolar bone in a similar distribution.

**Table 1:**
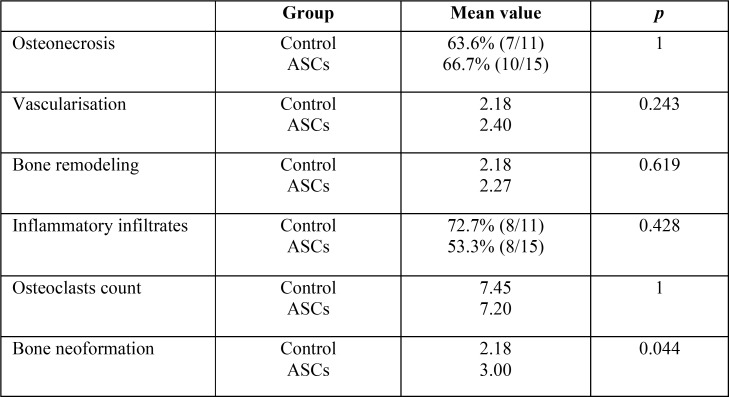
ASC versus control rats results.

**Figure 2:**
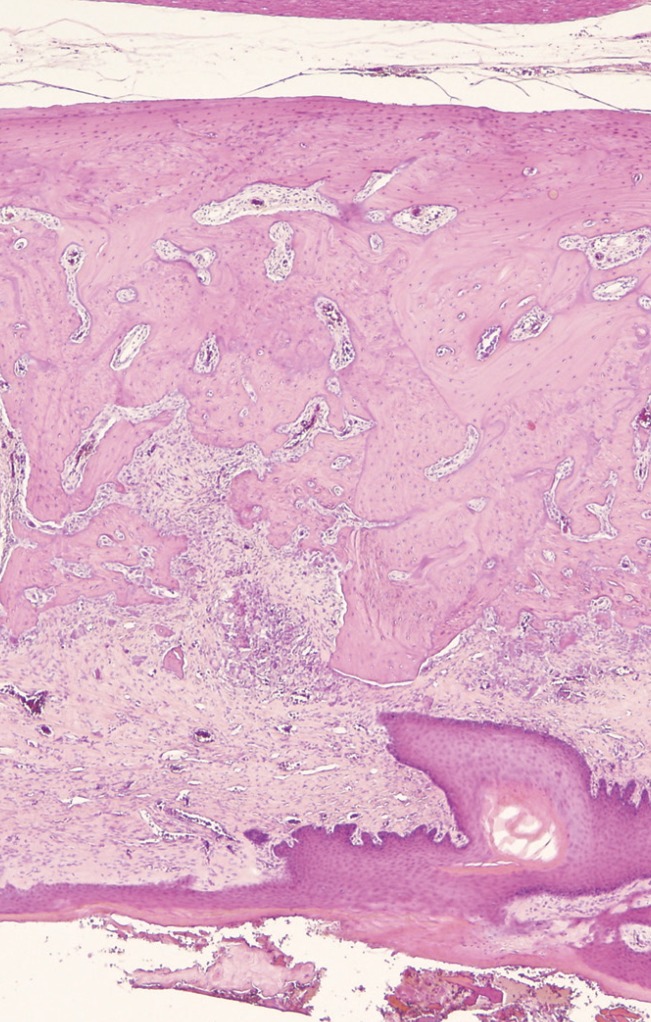
Histologic image of extraction sockets (haematoxylin-eosin, 4x). Control specimen (group 1). Little bone neoformation is observed.

**Figure 3:**
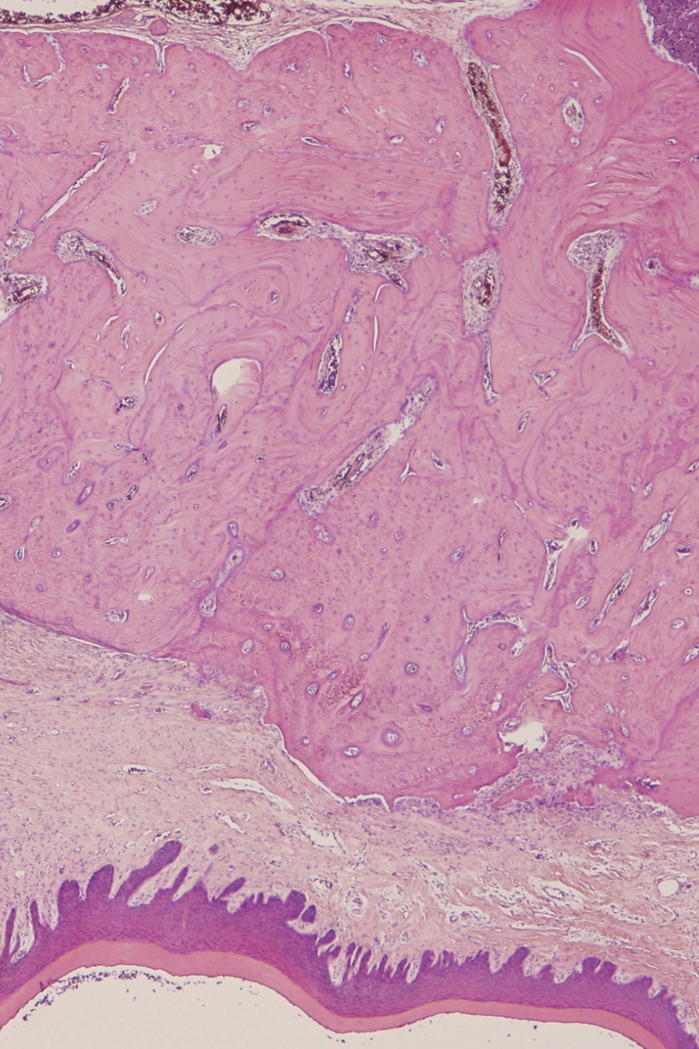
Histologic image of extraction sockets (haematoxylin-eosin, 4x). ASC-treated specimen (group 2). Greater new bone formation is observed in the treated specimen.

**Figure 4:**
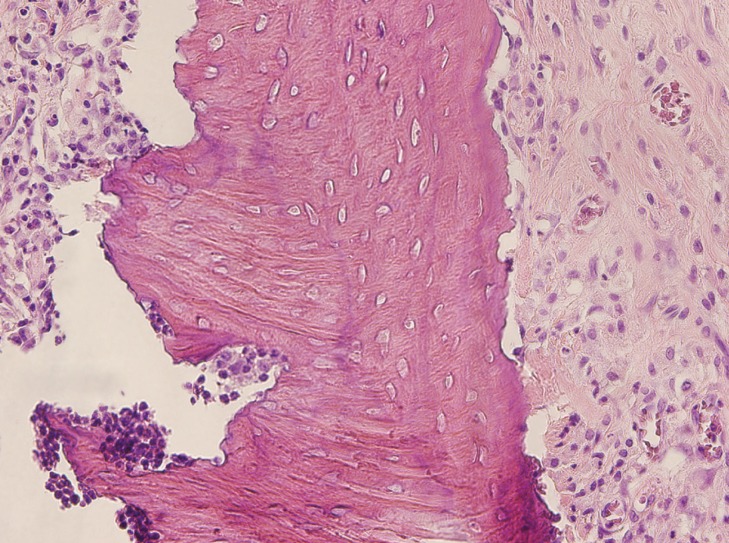
Histologic image of osteonecrosis (haematoxylin-eosin, 40x). Control specimen (group 1). Empty osteocyte lacunae are observed.

In the ASC-treated group, new bone formation was statistically significantly greater (with a mean value of 3.00 in the treatment group vs. 2.18 in the control group; *p*=0.044) (Figs. 2-4).

Vascularisation showed a greater mean value in the ASC-treated rats compared with the control group without reaching statistical significance.

No differences were observed in the remaining variables (inflammatory infiltrates, osteoclast count and bone remodelling).

-ASC vs. control rats euthanised 1 month after tooth extraction

Differences in groups 1 and 2 (treated and control rats euthanised 1 month after dental extractions) were analysed ( Table 2).

**Table 2:**
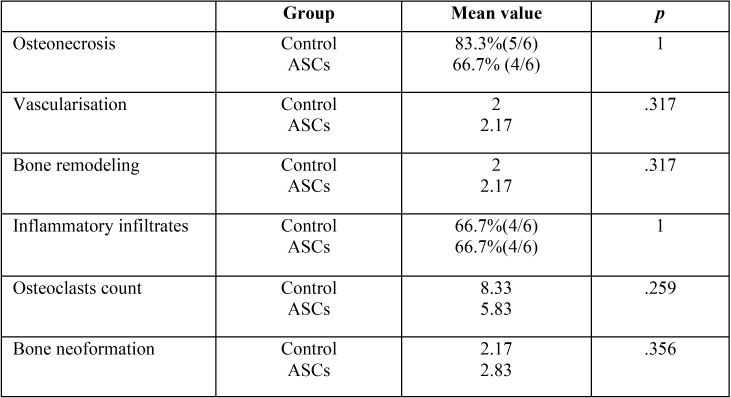
ASC versus control rats euthanised 1 month after tooth extraction.

Vascularisation and bone formation had greater mean values in the ASC-treated rats than in the control group, though statistical significance was not reached.

No statistically significant differences were found in osteonecrosis, osteoclasts, bone remodelling or inflammatory infiltrates.

-ASC vs. control rats euthanised 2 months after dental extraction

Vascularisation and bone formation showed greater mean values in the ASC-treated rats versus the control group, though without reaching statistical significance ( Table 3).

**Table 3:**
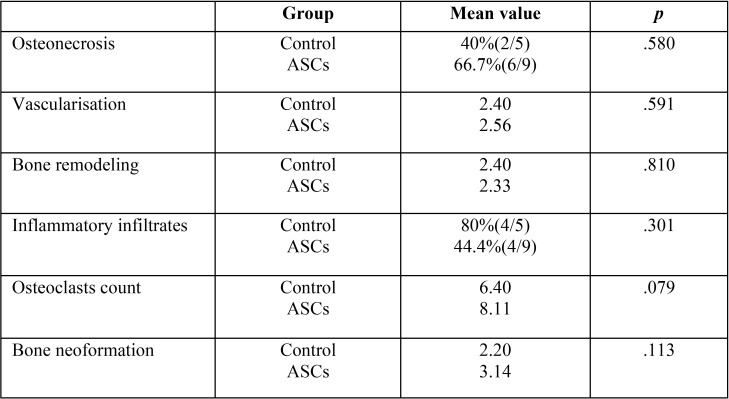
ASC versus control rats euthanised 2 months after tooth extraction.

Osteoclast numbers were greater in group 4 than in group 3, with a tendency towards statistical significance.

No statistically significant differences were found in osteonecrosis, bone remodelling or inflammatory infiltrates.

## Discussion

Currently, there is no standard BRONJ animal model due to a high degree heterogeneity of models (BP type, dosage, concomitant treatments…). In this study, osteonecrosis was induced after chronic administration of ZA, based on previous results(13) without other drugs to evaluate the real effect of BP treatment, with tooth extraction being the trigger for BRONJ-like lesions. As prevention strategies are essential in MRONJ, the potential preventive effect of locally applied ASCs on bone remodelling and regeneration of tooth extraction sockets has been evaluated.

None of the animals showed macroscopic bone exposure at the extraction sites. It is important to note that the unexposed bone stage of MRONJ (stage 0) in the AAOMS classification for humans, defined as clinical and radiological findings without bone exposure, nowadays appears to be a recognized stage (1). Despite the absence of bone exposure, histological features of osteonecrosis have been observed. When all the ASC-treated rats and all the control rats were compared, the presence of empty lacunae in alveolar bone was observed in a similar distribution. Osteonecrosis is similarly in both groups (in 66.7% of the specimens in the ASC group and 63.3% in the control group). Other authors such as Kobayashi *et al.* (17) didn’t observe bone exposure either. In their murine model, 250 µg/Kg/day of ZA was injected from 7 days before tooth extraction to 4 days after extraction. Although no macroscopic changes were observed in Kobayashi´s model, new bone formation in the tooth extraction socket was suppressed in mice treated with ZA. Matsumoto *et al.* (18) observed a deficiency of bone formation and remodelling but no bone exposure in aged rats with a more prolonged but inferior dose of ZA (0.035 mg/kg every 15 days).

Alveolar coverage with a mucoperiosteal flap, surgical milling of alveolar bone, the high regeneration capacity of the rat gingiva and the lack of other osteonecrosis enhancer drugs could have contributed to the absence of macroscopic bone exposure.

It was necessary to cover the alveolar sockets with a mucoperiosteal flap to contain the cells, and this step could have promoted healing, prevented contamination and decreased the risk of bone exposure. Lody *et al.* (19) and Heufelder *et al.* (20) recommend this measure in their protocols to prevent development of BRONJ after tooth extraction. Surgical milling of alveolar bone was carried out with a tungsten bur with the intention of adding more trauma. This manoeuvre possibly acted as an alveolectomy. It has been suggested that alveolectomy might favour complete healing of the extraction wound and decrease the risk of the occurrence of MRONJ(21).

In many BRONJ-like murine models, open wounds with bone exposure is only observed when BP is combined with corticoids (22,23) or chemotherapeutic therapy(11). In the present study, only ZA was administered to avoid confounding factors in the outcomes.

BPs inhibit the action of osteoclasts and induce apoptosis. This process leads to decreased bone resorption and remodelling. When osteoclastic action does not occur, the necessary steps for the activation of osteoblasts does not take place (24). An important finding in this study is that in the ASC-treated group, new bone formation was greater and statistically significant (see Table 1) and was always greater in the ASC subgroups independent of the time of evolution after tooth extraction. This discovery is important because it could be attributed to the effect of stem cells.

No differences were found in the number of osteoclasts when comparing both groups. However, the treated rats euthanised 2 months after tooth extraction showed a greater mean value than the control group, with a tendency towards statistical significance. This difference could also be due to the effect of the stem cells applied.

The increase in bone regeneration and bone remodelling could be attributed to the capacity of ASCs to differentiate directly into multiple mesodermal cell types (25), as well as into cells of ectodermal and endodermal lineages (26,27). However, according to findings in the literature, it is more probable that these results were related to the paracrine effects induced by ASCs. Lee *et al.* (28) have shown that ASCs could express and secret various cytokines, growth factors and proteins that are required for bone function and remodelling like M-CSF, RANKL, BMP-2, BMP-4, HGF.

Inhibition of angiogenesis is one of the hypotheses on the pathophysiology of osteonecrosis. ZA has shown significant antiangiogenic activity in several in vitro and in vivo models through a reduction in serum VEGF (Vascular endothelial growth factor) levels (29,30). In addition, it has been demonstrated that ASCs secrete multiple proangiogenic growth factors as VEGF(31). Vascularisation showed a greater mean value in the ASC-treated rats in all groups, though statistical significance was not reached.

Adipose tissue has been used in this model because it is an abundant and easily accessible source of stem cells. In addition, the procedure to obtain ASCs is a safe and minimally invasive procedure, associated with low discomfort for patients. Although BM-MSCs and ASCs share many properties, they also present some differences. Some studies suggest that BM-MSCs could be more prone to osteogenic differentiation than ASCs *in vitro* (32,33), but other authors don’t find any differences (34,35). ASCs seems to have a higher immunomodulatory capacity than BM-MSCs (36,37) which could improve immune dysfunction related to BPs (4,6).

There are few reports about stem cells and MRONJ and most of them study the treatment effects but not the preventive effects of ASCs.

Some studies on MSCs in the treatment of this condition are the reports of Kaibuchi *et al.* (3), Kikuiri *et al.* (4) and Ogata *et al.* (5), which have shown that treatment with BM-MSCs is effective for the treatment of osteonecrosis-like disease in murine models. ZA and dexamethasone had been administered in these reports to generate the BRONJ-like model. Li *et al.* (6) observed bone exposure with BP-only treatment in larger animals (minipigs), and showed new bone formation in previously necrotic areas after BM-MSC infusion. There are a few reports on humans with good results, but with a low level of evidence (7-9). All of these studies use MSCs isolated from bone marrow.

Stem cells have been recently investigated as a preventive factor in MRONJ. Kikuiri *et al.* (4) showed that systemic infusion of BM-MSCs prevents and cures BRONJ-like disease in a mouse model via induction of peripheral tolerance. Kuroshima *et al.* (11) investigated the effects of non-cultured stromal vascular fraction (SFV) transplantation on tooth extraction socket healing in mice. Specimens were treated previously with chemotherapeutic/bisphosphonate combination therapy and they stated that systemic transplantation of SVF cells ameliorates BRONJ-like lesions by improving both osseous and soft tissue healing of tooth extraction sockets. The SVF is a heterogenous population including endothelial cells, pericytes, fibroblasts, preadipocytes, ASCs... (25,38), so they have different properties compared with purified ASCs.

With the objective of eliminating possible confounding factors and analysing only the effects of ASCs in the present study, stimulation with bone morphogenetic protein 2 and platelet-rich plasma have been eliminated from our research group’s previous model (10). In the present model using ASCs only, new bone formation was greater in the treated group, suggesting that previously inhibited post-extraction bone remodelling could be reactivated. There is also bone formation in the control group, but it is significantly less and since the only difference between groups is the implantation of ASCs, these findings appeared to be secondary to the effects of stem cells.

Further studies are needed to confirm our preliminary results on ASCs as a preventive strategy in MRONJ, but these results appear to be promising.

Prevention strategies are essential in MRONJ and this field needs to be investigated. In this study, the effect of ASCs in an experimental murine model of osteonecrosis was analysed and the treated group has shown greater new bone formation. These results are promising and establish a basis for further studies and subsequent clinical trials in humans on the effect of ASCs as a preventive factor for this pathology.
